# Questionable indication for postoperative radiotherapy after surgery for metastases of the major long bones: A single-centre cohort study of 552 fractures/526 patients

**DOI:** 10.1016/j.jbo.2026.100746

**Published:** 2026-02-05

**Authors:** Jessica Ehne, Adele Kastensson, Rikard Wedin, Panagiotis Tsagkozis

**Affiliations:** a Department of Molecular Medicine and Surgery, Karolinska Institutet, Stockholm, Sweden; b Department of Orthopaedic surgery, Karolinska University Hospital, Stockholm, Sweden

**Keywords:** Bone neoplasms/secondary, Bone neoplasms/ therapy, Radiotherapy, Adjuvant, Metastatic bone disease, Bone metastases

## Abstract

**Introduction:**

The use of postoperative radiotherapy (PORT) as an adjunct to orthopaedic surgery of the long bones is widely recommended. Evidence of the benefits is however sparse. The primary research question of this study was whether PORT reduces the incidence of local failure after orthopaedic surgery for metastatic disease of the long bones.

**Method:**

A prospectively collected database was retrospectively reviewed. A total of 552 fractures/526 patients were included. Primary endpoint was secondary surgery due to implant failure due to local tumour progression. The main analysis used a competing risk model with death as a competing event.

**Results:**

PORT was administered in 197 cases, the median initiation time was 42 days and the most common schedule was 4 Gy × 5. 33 cases (6%) required revision attributed to tumour related implant failure. The cumulative incidence of failure was higher in the PORT group (p = 0.038). When dividing cases into subgroups depending on surgical method PORT had no effect in the osteosynthesis group and was significantly associated with a worse outcome in the prosthesis group. PORT dose and timing were not associated with failure rates.

**Conclusion:**

In this cohort, PORT was associated with a significantly increased risk of tumour related failure in patients treated with a prosthesis. There was no significant effect in the osteosynthesis subgroup. Our results indicate that there is no clear evidence for the universal use of PORT.

## Highlights

• Postoperative radiotherapy was not associated to lower tumor-related implant failure after surgery for metastasis in major long bones.
• Postoperative radiotherapy was associated with increased revision risk in patients treated with prostheses.
• Findings question routine use of radiotherapy after surgery of the major long bones and suggest it should be used only in selected cases.

## Graphical abstract

**Figure:**
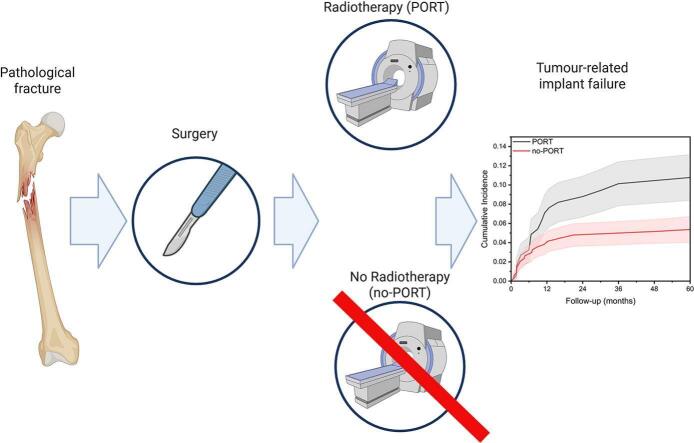

## 1 Introduction

As the population ages and patients with cancer are living longer, the incidence of metastatic bone disease (MBD) is expected to rise [1]. Patients with MBD who develop impending or actual fractures have poor survival outcomes [2]. Bone metastases are painful and negatively affect quality of life [3], [4], [5]. Although most bone metastases affect the axial skeleton, the femur and humerus with their rich bone marrow are the most common MBD sites in the appendicular skeleton [5].

Radiotherapy (RT) is used to reduce pain and tumour bulk in MBD. However, when a patient presents with an impending or manifest fracture, surgery is considered first line treatment [5], [6], [7], [8]. In these cases, surgery is technically easier than in the axial skeleton and it is often possible to reduce tumour bulk extensively. Two main surgical approaches are used: stabilization of the remaining bone with an osteosynthesis (often combined with tumour debulking and cementation) or replacement of the affected bone segments with a prosthesis. Factors influencing the surgical approach include both bone related aspects such as fracture site and remaining bone stock as well as patient related factors such as general condition and comorbidities, the latter two strongly influencing postoperative survival [2], [8], [9], [10], [11]. Both surgical methods are often combined with cementation, of voids or the implant itself. The use of bone cement, polymethylmethacrylate (PMMA) may also provide local tumour control [12].

Postoperative RT (PORT) is used as an adjuvant treatment to orthopaedic surgery and is common clinical practice worldwide. It is thought to reduce tumour burden, slow tumour growth, and minimize microseeding from reaming through the tumour. PORT is also used to reduce pain and the risk of implant loosening through the reduction of local tumour growth and thus preventing implant failure, a clinical catastrophe for these, on a group level, very frail patients [7], [13], [14].

Despite being widely used and theoretically appealing, evidence on the clinical practice of PORT is weak, relying primarily on small retrospective cohort studies or case series reporting on surgical outcomes and tumour growth in various ways [14], [15], [16], [17], [18], [19], [20]. This practice has been challenged [21], and to our knowledge no subsequent studies have provided definitive support, though some authors have noted trends toward reduced local tumour progression and implant failure in patients receiving PORT [15], [16], [17], [18], [19], [20].

With this study, our primary research aim was to assess whether PORT, delivered at the standard doses and fraction schemes used at our institution, provided clinical benefit in terms of reducing the risk of reoperations and implant failure in patients undergoing surgery for MBD. As RT can damage tissue and could also lead to an increase in infection rates [22], our secondary aim was to analyse the impact of RT, both preoperative and PORT on postoperative infections.

## 2 Materials and methods

All included patients were retrieved from the prospectively collected databases of the institution and processed anonymously in accordance with local ethical guidelines (ethical permits 2012/272–31 and 2019–06189 of the Regional Ethics Committee). Data regarding postoperative RT, surgical method and complications were cross checked and missing data completed retrospectively from patient electronic files by 1st, 2nd and 3rd author.

Inclusion criteria: Primary surgery between January 2000 and June 2024. Surgery had to entail the use of an implant, osteosynthesis or prosthesis and the anatomic location had to be the humerus or femur MBD from solid cancers and haematological malignancy were accepted, primary bone cancers were excluded. Out of county referrals were also excluded.

When nails were used, there was no significant debulking of the tumour. There was no cementation when nails were used. When plates were used, the lesion was usually curetted to debulk the tumour. In cases of prosthesis, curettage was performed before cementing the implant, except for in 53 cases which had surgery with en bloc excision and megaprosthetic reconstruction.

The medical records were reviewed regarding surgical method (osteosynthesis or prosthesis), surgical complications and the use of pre- and post-operative radiotherapy including date and dose. Radiotherapy at 6 months or later after surgery was considered irrelevant to the surgical outcome, radiotherapy to the operated field within six months after surgery was considered as PORT. Separate analysis was made considering PORT as radiotherapy administered within 6 weeks or 3 months. Tumours were classified according to their radiosensitivity (radiosensitive: haematological malignancy and small-cell lung cancer, moderately radiosensitive: breast cancer and squamous cell carcinoma, moderately radioresistant: prostate cancer, lung adenocarcinoma, colon adenocarcinoma, radioresistant: melanoma, sarcoma, renal cell carcinoma, thyroid and hepatocellular cancer). Local failure due to tumour progression was defined as the primary endpoint. Tumour progression was determined based on the clinical symptoms, and verified radiologically using conventional radiography. Radiographs were assessed by two radiologists and the orthopaedic surgeon who performed the surgery. Failure was defined as secondary surgery entailing change or removal of any part of the implant. Patients not undergoing revision surgery were classified as non-events. Revision surgery due to mechanical failure and infection were analyzed separately. Revision surgery due to mechanical failure and infection were also analyzed separately, as secondary endpoints. Mechanical failures related to surgical errors were not considered relevant to the aim of this study.

Statistical analysis was done in the SPSS (version 25, SPSS Inc, Chicago, IL) and the SAS statistical package (SAS University Edition). Data from SAS were plotted in graphs using OriginPro 2024 (OriginLab Corporation). Pearson’s chi-square (χ2) test was used for comparisons between groups. Patient survival analysis was done using the Kaplan-Meier method, and comparison between groups using the log-rank test. Given the presumed high early postoperative mortality, a competing risks survival analysis regarding implant survival was done using the Fine and Gray’s model. Multivariable proportional hazards regression with the as the competing risk event was also performed. All tests were double-sided, and a p value of ≤0.05 was considered significant.

## 3 Results

**Fig. 1:**
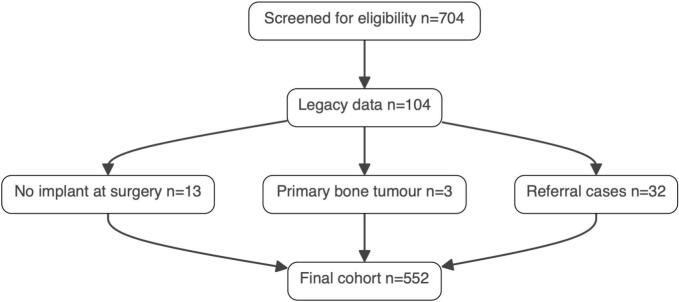
Final cohort consisted of 552 cases after removal due to preset inclusion criteria. In the reviewed cases, 552, we had 526 patients. There was missing data regarding generalized/single tumour in 2 cases, fracture type humerus/femur 4 cases and preoperative radiotherapy in 2 cases.

A total of 526 patients underwent 552 surgeries (Fig. 1). Median patient survival was 201 days/6.6 months, mean: 485 days/16 months. Medial follow-up time was 81 months. Mortality was 11% at 1 month and 30% at 3 months. One-year survival was 36%, and 21% survived for two years or more. Most patients had intralesional surgeries with a conventional prosthesis or osteosynthesis. 10% (53/552) had surgery with a tumour specific prosthesis. Median and mean age was 71 years. The cohort included 264 males (50%) and 262 females (50%). Solitary metastases were present in 65 patients (12%), and multiple metastases in 461 patients (88%). Most patients had haematological malignancy (111), breast cancer (110), lung cancer (91) or prostate cancer (84), 47 patients were diagnosed with kidney cancer.

PORT was administered in 197/552 cases. The decision to offer PORT was individualized and made by the treating oncologist after consultation with the patient. In patients not referred to or declined PORT, 84% were due to general deterioration in their underlying cancer disease. 8% were already at or close to the maximum RT limit. The remaining patients were thought to have infections (but most of these had PORT once the infection was treated) or there was no mention of the reason in the charts.

Corroborating multiple published studies, patients with prosthesis had a superior overall survival (p = 0.01), whereas femoral prostheses displayed a significantly lower tumour-associated revision rate as compared to osteosyntheses (p = 0.003).

The mean and median time from surgery to initiation of PORT was 6 weeks (range, 2–181 days). The mean and median radiotherapy doses were both 20 Gy (range 8–52 Gy) with a schedule of 4 Gy times 5 being the most common treatment plan. Data on the modes of radiotherapy are provided in Supplementary Table 1.

Table 1 descriptive statistics and information on subgroups related to administration of PORT.

**Table 1:** Cohort characteristics and association with receipt of postoperative radiotherapy (PORT) by Pearsons chi-square test. Patients who had their implants cemented or cement added to the bone and patients with multiple metastases had significant P-values when compared to those with no added cement and patients with single metastases. Confidence intervals however cross 1 for both variables. In this cohort male patients were more likely to receive PORT than female patients.

Variable	Category	PORT (n)	noPORT (n)	p-value
Gender	Male	121	143	
	Female	76	212	<0.001
Metastases	Solitary	14	47	
	Multiple	182	305	0.027
Fracture	Complete	142	261	
	Impending	55	92	0.64
Cemented	Cemented	118	179	
	Uncemented	79	176	0.032
Method	Prosthesis	67	137	
	Osteosynth.	130	218	0.29
Location	Femur	171	294	
	Humerus	26	61	0.22
Age (mean, years)	65	66	0.583	
Major Diagnoses	Haematological 31	80	0.029	
	Breast	36	75	0.021
	Lung	35	56	0.56
	Prostate	32	52	0.19
	Renal	22	25	0.076

88 patients required return to surgery for any cause, including closed reduction. Among these, 36 patients underwent more than one surgical procedure. In total, 67 patients had their implants either removed or revised, 37 of these were attributed to tumour related reasons. Additionally, 16 patients experienced such clinical deterioration that revision surgery was deemed inappropriate.

There was no difference in revision due to tumour related reasons between late (>42 days) and early PORT (<42 days) groups (p = 0.635).

**Fig. 2:**
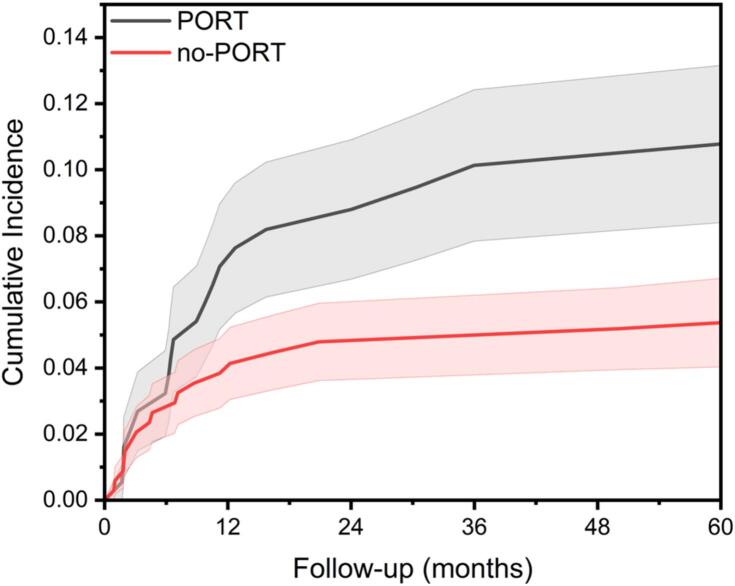
Cumulative incidence of implant failure due to tumour growth over follow-up time (in months) for two groups: patients who received PORT, and those who did not. Shaded areas around each curve represent the 95% confidence intervals. There was no significant difference between groups p = 0.038, but a trend towards higher risk of failure in the PORT group.

**Fig. 3:**
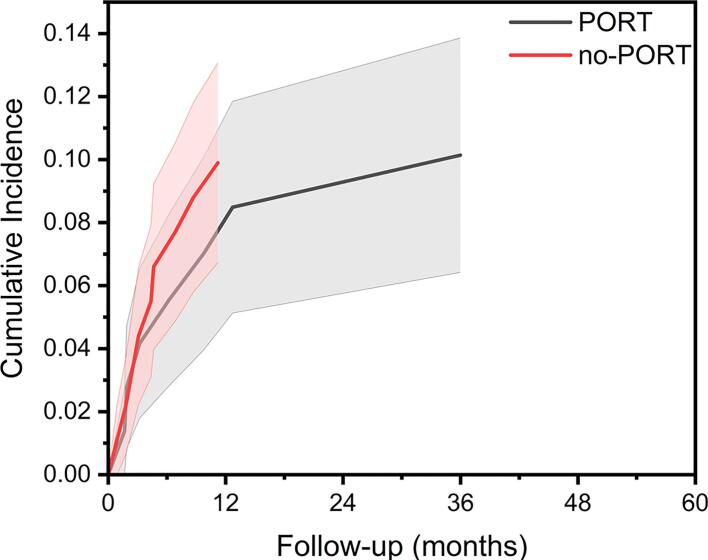
Cumulative incidence function of tumor growth in the osteosynthesis subgroup, stratified by PORT status, compared to no-PORT. Shaded regions represent 95% confidence intervals. There was no difference between groups, p = 0.97.

**Fig. 4:**
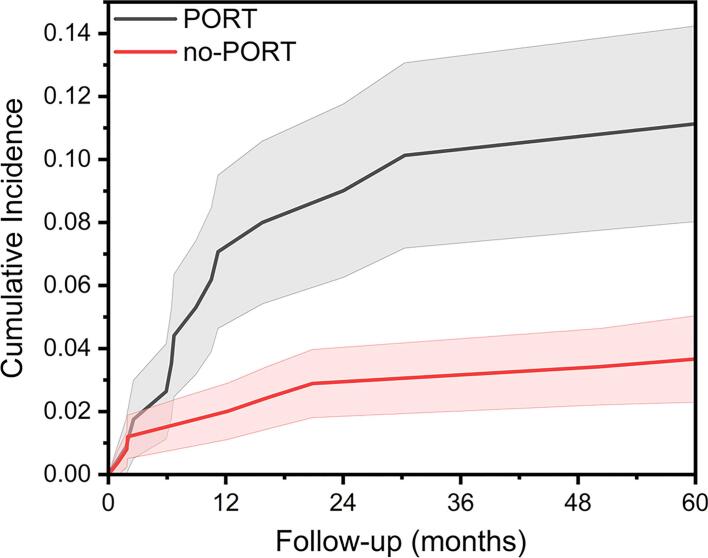
Cumulative incidence function of tumor growth in the prosthesis subgroup, stratified by PORT status compared to no PORT. Shaded regions represent 95% confidence intervals. PORT group had higher cumulative incidence of implant failure. P = 0.01.

When using death as a competing risk, there was a higher risk of revision when receiving PORT (p = 0.038) and especially in cases operated with a prosthesis, p = 0.01. When the surgical method was an osteosynthesis there was no statistical difference (p = 0.97). See Supplementary Table 2 and Fig. 2, Fig. 3, Fig. 4. When the cut-off for PORT was set to 6 weeks or 3 months after the index surgery, there was no significant association between PORT and tumour related failure, in either prostheses (p = 0.06) or osteosyntheses. p = 0.11) In a multivariable regression analysis with death as a competing risk event, PORT was not associated to tumour-related failure (Supplementary Table 3). Neither was there a significant association of PORT to tumour-related failure when the analysis was performed for femoral (p = 0.851) and humeral (p = 0.462) lesions separately, or when performed only for patients that had survived more than 3 months free of failure (data not shown). Finally, when we restricted our analysis to radiosensitive tumours, there was no significant association of PORT to implant failure rate (p = 0.06), although the general trend that failure was more likely to be seen in patients who received PORT was also observed.

Preoperative radiotherapy was administered in 118/550 cases (2 missing cases), mean time to surgery was 180 days, median time to surgery was 103 days. 13 patients had both pre and postoperative RT. Preoperative radiation therapy was not significantly associated with a higher risk of infection, p = 0.162 (CI 0.84–3.10).

There were 40 postoperative infections, 19 leading to revision. Of patients treated with PORT, 4% had a superficial or deep infection compared to 10% in the no-PORT group. Patients receiving PORT had a significantly lower incidence of postoperative infection, p = 0.007.

## 4 Discussion

To our knowledge, this is the largest consecutive cohort to specifically examine the effect of PORT on revision surgery, excluding surgery due to infection or technical errors, in long bone metastases [14], [15], [16], [17], [18], [19], [20]. As previously recommended, we prespecified the use of a competing risk analysis with death as a competing event to avoid biased conclusions from short patient survival times [20]. In our cohort 36% lived past the first year following surgery with prior studies reporting implant failures typically occurring at 12–24 months postoperatively [10], [19] we believe that the suggestion from other researchers to use death as a competing risk holds true.

Unexpectedly our findings suggest a possible hazard associated with PORT, particularly in prosthesis cases. A potential explanation for the lack of observed benefit from PORT in our cohort, besides the short patient survival, is the high proportion of cemented implants. Since bone cement is known to generate cytotoxic heat during polymerization, which may exert a tumoricidal effect on residual tumour cells within the bone, potentially making PORT redundant [12]. Additionally, our median interval between surgery and the initiation of PORT was 42 days, which is longer than in prior studies that emphasized the importance of early radiotherapy [14], [16], [18]. Because of this we performed additional analysis evaluating PORT administered within 6 weeks and 3 months which did not alter results, still showing a trend towards worse outcome in the PORT prosthesis group although unsignificant (p = 0.006 and p = 0.064). Another methodological consideration was the classification of revision surgeries. Failures were classified according to the predominant mode of failure, reflecting clinical practice. To address the potential impact of this approach, we also performed an additional analysis including all failures except those attributable to surgical error. This analysis similarly showed no association between PORT and a reduced failure rate, supporting the robustness of the primary findings. In a similar way radiosensitivity was also considered and separately analyzed for radiosensitive tumours, defined as small cell and haematological malignancy, this subgroup analysis did not show any benefit of PORT (p = 0.01 for osteosynthesis and p = 0.89 for prosthesis) either.

We did also have a large proportion of patients with hematologic malignancy in our cohort, the majority with multiple myeloma, a slow growth cancer type with good systemic response, which could mitigate PORT-effect.

Our intention was to examine the use of PORT in the clinical setting. Our findings reflect real world clinical practice and are also supported by previous cohort studies not being able to demonstrate any statistical difference in implant survival between PORT-no-PORT groups. [18], [20], [21].

In this cohort, PORT was associated with a lower rate of postoperative infection, obviously reflecting selection bias. Our results support current guidelines [6], [7] that PORT is safe once the wound has healed, they also demonstrate that clinicians are able to make this clinical decision without putting the patient at risk. PORT should not be withheld due to concerns regarding infection were there are no such clinical signs.

Limitations: Our study has all the limitations associated with the nature of retrospective studies. A major methodological limitation is the accuracy of failure classification, since microscopic disease may have contributed to failure classified as non-tumour related. However, multiple analyses of other failures showed no association to PORT either. At the same time, we acknowledge that the low number of primary endpoint events means that the statistical power of our study is low despite the large number of patients included. This in turn highlights that failure due to tumour progression is a quite rare event, and interventions may have a high expected number of patients treated to observe a meaningful result. Our data compared to that of previous reports indicate a higher return to surgery [15], [16] and a lower postoperative survival [9], [20] indicating that as a single centre study our outcomes may not be directly applicable to other hospitals or countries.

## 5 Conclusion

This study included 552 consecutive surgical procedures in 526 patients prospectively registered at our institution. Our primary aim was to analyze the efficacy of PORT in preventing revision surgery in MBD patients following orthopedic surgery of the major long bones. In our cohort there was no effect of receiving PORT on implant survival in patients operated with an osteosynthesis, in patients operated with a prosthesis receiving PORT was linked to an increased risk of implant failure.

As therapies not clearly adding to quality of life should be avoided when caring for palliative patients we would advise to use PORT only in selected cases. Typical indications would patients with a long expected remaining survival and operated with an osteosynthesis. Our study highlights the need for further research in the field, preferably as a prospective randomized trial.

Finances and conflicts of interests: The authors declare that they have no conflicts of interest relevant to the content of this study. This study was supported by institutional resources from the Department of Molecular Medicine and Surgery, Karolinska Institutet. No external funding was received.

Declaration of generative AI and AI assisted technologies in the manuscript preparation process: Chat GPT 5.0 was used for language editing. Authors take full responsibility.

## CRediT authorship contribution statement

**Jessica Ehne:** Writing – original draft, Visualization, Validation, Investigation, Formal analysis, Data curation. **Adele Kastensson:** Writing – review & editing, Formal analysis, Data curation. **Rikard Wedin:** Writing – review & editing, Supervision, Data curation. **Panagiotis Tsagkozis:** Writing – review & editing, Writing – original draft, Validation, Supervision, Project administration, Methodology, Conceptualization.

## Declaration of competing interest

The authors declare the following financial interests/personal relationships which may be considered as potential competing interests: Panagiotis Tsagkozis reports administrative support and writing assistance were provided by Karolinska Institute. If there are other authors, they declare that they have no known competing financial interests or personal relationships that could have appeared to influence the work reported in this paper.
