# Supplementary material for: Questionable indication for postoperative radiotherapy after surgery for metastases of the major long bones: A single-centre cohort study of 552 fractures/526 patients
Source: J Bone Oncol. 2026 Feb 5;57:100746. doi: 10.1016/j.jbo.2026.100746 (PMC12907229; doi:10.1016/j.jbo.2026.100746)
Supplement: Supplementary Data 1 [file mmc1.docx]

Supplementary table 1

| **Treatment course** | **Fractionation schedule** | **Total dose (Gy)** | **N** |
| --- | --- | --- | --- |
| **Single-fraction** | 8 Gy × 1 | 8 | 33 |
|  | 20 Gy × 1 | 20 | 1 |
| **Short-course** | 4 Gy × 5 | 20 | 78 |
|  | 8 Gy × 2 | 16 | 42 |
|  | 5 Gy × 5 | 25 | 19 |
|  | 4 Gy × 6 | 24 | 3 |
| **Long-course** | 3 Gy × 10 | 30 | 17 |
|  | 3 Gy × 13 | 39 | 2 |
|  | 20 Gy × 2 | 40 | 1 |
|  |  |  | 196 |

### **Supplementary Table 2. Gray's Test for Equality of Cumulative Incidence Functions Results**

| **Cohort** | **Chi-square** | **p-value** |
| --- | --- | --- |
| **Overall** | 4.2965 | 0.0382 |
| **Osteosynthesis** | 0.0012 | 0.9720 |
| **Prosthesis** | 6.6319 | 0.0100 |

*Supplementary table 3,* multivariable regression analysis

| **Variable** | **Category** | **HR** | **p-value** |
| --- | --- | --- | --- |
| Tumor spread | Multiple skeletal/single metastasis | 5.1 × 10⁷ | <0.0001 |
|  | Visceral metastases/single metastasis | 9.3 × 10⁷ | <0.0001 |
| Tumor location | Femur vs humerus | 0.05 | 0.0012 |
| PORT | Yes/no | 5.07 | 0.099 |
| Gender | Male/female | 0.999 | 0.999 |
| Cementation | Yes/no | 0.34 | 0.48 |
| Age | Per year increase | 1.01 | 0.512 |
|  |  |  |  |
